# A Population Proportion approach for ranking differentially expressed genes

**DOI:** 10.1186/1471-2105-9-380

**Published:** 2008-09-18

**Authors:** Mugdha Gadgil

**Affiliations:** 1 Chemical Engineering and Process Development, National Chemical Laboratory, Pune, 411008, India

## Abstract

**Background:**

DNA microarrays are used to investigate differences in gene expression between two or more classes of samples. Most currently used approaches compare mean expression levels between classes and are not geared to find genes whose expression is significantly different in only a subset of samples in a class. However, biological variability can lead to situations where key genes are differentially expressed in only a subset of samples. To facilitate the identification of such genes, a new method is reported.

**Methods:**

The key difference between the Population Proportion Ranking Method (PPRM) presented here and almost all other methods currently used is in the quantification of variability. PPRM quantifies variability in terms of inter-sample ratios and can be used to calculate the relative merit of differentially expressed genes with a specified difference in expression level between at least some samples in the two classes, which at the same time have lower than a specified variability within each class.

**Results:**

PPRM is tested on simulated data and on three publicly available cancer data sets. It is compared to the t test, PPST, COPA, OS, ORT and MOST using the simulated data. Under the conditions tested, it performs as well or better than the other methods tested under low intra-class variability and better than t test, PPST, COPA and OS when a gene is differentially expressed in only a subset of samples. It performs better than ORT and MOST in recognizing non differentially expressed genes with high variability in expression levels across all samples. For biological data, the success of predictor genes identified in appropriately classifying an independent sample is reported.

## Background

DNA microarrays are used to monitor the expression level of thousands of genes simultaneously, and are extensively used in various areas of biological research [1-4]. The reader is referred to Schena [5] and Bowtell and Sambrook [6] for a detailed introduction to microarray technology. A biological problem which is being increasingly addressed through the use of microarray assays is the identification of differences in gene expression between two or more classes of samples e.g. between disease and normal tissue [7-18]. The methods for identifying differentially expressed genes vary greatly [19-27], but all have a goal of identifying genes with a significant difference in expression level between samples in the two classes. A simple method to analyze such data is to compare the sample means of the expression level of each gene in the two classes to obtain a 'fold-change' [28] in the expression level of the gene between the two classes. However, fold change calculations fail to account for variability in expression levels between samples within a class. As aptly pointed out by Simon *et al *[29], "some twofold average effects represent statistically significant differences and some do not". Statistical methods like t-test [30,31] and ANOVA [32-34] are used to assess the significance of differential expression by incorporating data on variability between samples. Many alternative approaches of incorporating data on variability have also been developed [19-21,26,27,35].

Unlike the case of replicate *in vitro *data which are expected to have extremely low intra-class variability under ideal conditions, the expression level of a gene can vary significantly within samples obtained from different individuals in one class due to biological variation [36]. Also, clinically similar phenotypes can be caused by different molecular mechanisms [37]. Genes which are differentially expressed in only a subset of samples in a class can be important in such cases [38-40]. Most analysis methods compare the means of intra-class expression levels and are not likely to find genes whose expression is significantly different in only a subset of samples in a class, or have high intra-class variability.

A few approaches have been previously proposed to identify such genes [38,39,41-43]. One approach to identify such genes proposed by Lyons-Weiler *et al *[39], is the Permutation Percentile Separability Test (PPST). This test identifies genes for which a statistically significant number of samples in group A exhibit expression intensities beyond a particular percentile of the observed expression intensities of that gene in group B. Another approach is proposed by Bijlani *et al *[38] who compare the expression level of a gene in every sample in one class to the mean of the expression level in the other class. The proposed application of this method is to select genes which can be used for class distinction. Tomlins *et al *[42], Tibshirani *et al *[41], Wu *et al *[43] and Lian *et al *[44] use variations of transformation of gene expression values using the sample median and median absolute deviation in the Cancer Outlier Profile Analysis (COPA), Outlier sums (OS), Outlier Robust *t*-statistics (ORT) and Maximum Ordered Subset *t*-statistics (MOST) methods respectively. The performance of COPA and OS has been shown to deteriorate as the number of outliers increase [43].

All the methods listed above except PPST use some normalized form of the algebraic difference between expression levels as a measure of heterogeneity to identify 'outliers'. These methods might not be suitable for cases where a subset of samples in a class are responsible for significantly increasing the variability in the class, and are spread over a large range. Consider the following hypothetical example; a group of 10 samples have expression levels of a gene as [50, 50, 75, 80, 100, 120, 120, 300, 500, and 700]. Defining an outlier as a value more than the interquartile range above the third quartile, as used by some researchers [43], only one sample (700) is identified as an outlier. However a closer look at the data indicates that the last three samples are responsible for the increased variability in the class. This motivated the need to explore alternative ways to quantify variability.

**Figure 1:**
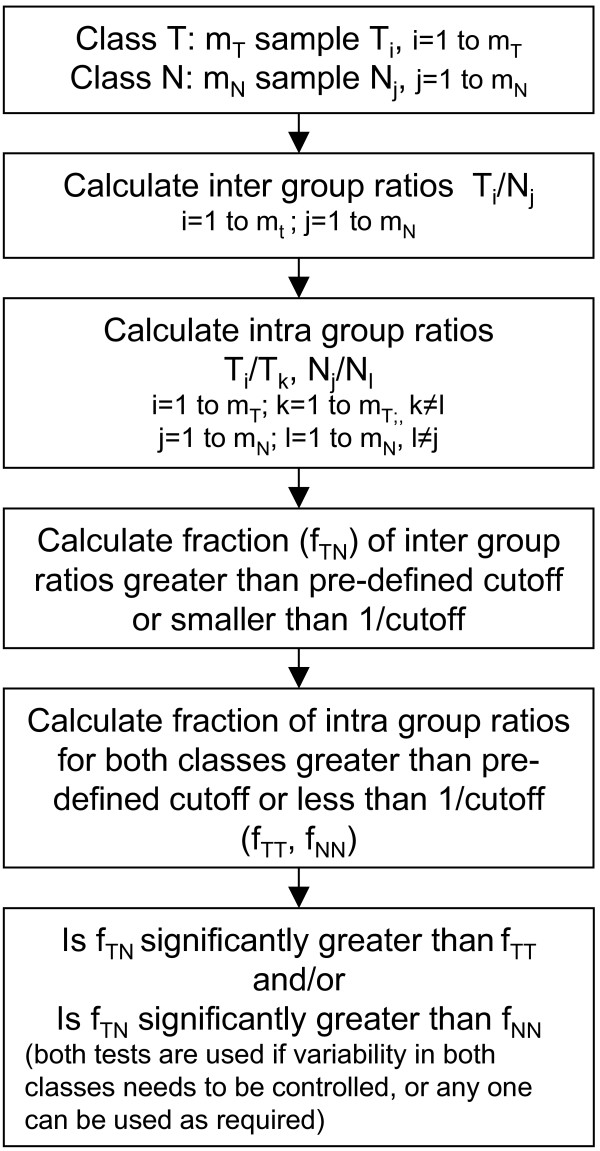
**Summary of the Population Proportion Ranking Method**. The inter-class variability is quantified by calculating the inter-class ratio of expression level of a sample in class T to its expression level in a sample in class N, for all possible combinations of samples in the two classes. Depending on the desired relative difference between the classes to identify a gene as differentially expressed, an inter-class ratio cutoff is chosen. The fraction of inter-class ratios calculated above, which are greater than this inter-class ratio cutoff, is calculated (f_TN_). Intra-class variability for a class is similarly quantified by calculating the intra-class ratios of expression level of a sample in the class to its expression level in every other sample in the same class. Analogous to the inter-class ratio cutoff, an intra-class ratio cutoff is chosen based on acceptable level of variability within a class. The fraction of intra-class ratios calculated above which are greater than the cutoff is calculated (f_TT_, f_NN_). Genes in which f_TT _and/or f_NN _fraction is significantly smaller than f_TN _are ranked based on an established statistical method of comparing population proportions.

This paper presents a Population Proportion Ranking Method (henceforth referred to as PPRM) to qualitatively rank differentially expressed genes. This method uses inter-sample ratios to quantify variability in expression levels. To my knowledge, this is the first reported method using this approach. The method allows the user to pre-define the required magnitude of difference in expression level of a gene between samples in the two classes and the allowable level of intra-class variability, and has the ability to identify genes which might be differentially expressed in only a subset of the samples in a class and have high variability within a class. The basic steps in the method are outlined in Figure 1. Briefly, the inter-class variability is quantified by calculating the ratio of expression level of a sample in class T (Treated) to its expression level in a sample in class N (Normal), for all possible combinations of samples in the two classes (referred to henceforth as interclass ratios). Depending on the desired relative difference between the classes to identify a gene as differentially expressed, an inter-class ratio cutoff is chosen. The higher the inter-class ratio cutoff, the greater the required difference between classes. The fraction of inter-class ratios calculated above, which are greater than this inter-class ratio cutoff, is calculated (f_TN_). A higher value of f_TN _implies that a larger proportion of samples have the required difference between the two classes.

Intra-class variability for a class is similarly quantified by calculating the ratios of expression level of a sample in the class to its expression level in every other sample in the same class (referred to henceforth as intra-class ratios). Analogous to the inter-class ratio cutoff, an intra-class ratio cutoff is chosen based on acceptable level of variability within a class. The fraction of intra-class ratios calculated above which are greater than the cutoff is calculated (f_TT _& f_NN_). Genes in which these fractions are significantly smaller than f_TN _are ranked based on an established statistical method of comparing population proportions [45].

Simulated data sets where the truly differentially expressed genes are known are used to test the ability of PPRM to identify differentially expressed genes. The performance of PPRM is compared to the t test, PPST[39], COPA [42], OS [41], ORT [43] and MOST [44] for the simulated data, and is found to be comparable or better under the conditions tested. Thus, PPRM could be a valuable addition to the repertoire of existing methods for detecting genes differentially expressed in a subset of samples in a class. However, simulated data sets do not necessarily mimic the variability in real biological data sets. Hence, this method is also applied to three publicly available cancer data sets to identify differentially expressed genes.

Since there is no gold standard of true differentially expressed genes in an experimental study, an approach of using differentially expressed genes identified by the method as predictors to test their ability to successfully classify independent sample(s) is used for validation of the method in real-world data. This approach was also used by Jeffery *et al *for evaluation of lists of differentially expressed genes identified [46]. The method proposed in this paper is tested on 3 publicly available cancer data sets: leukemia [18], colon cancer [47] and prostate cancer [48]. In case of the leukemia data set, an independent sample set is available to test whether the top differentially expressed genes identified can correctly classify independent samples. For the other two data sets, leave-one-out cross-validation (LOOCV) is implemented to test the accuracy of classification.

The particular method of choice for identifying differentially expressed genes depends on the biological question, and PPRM provides an additional tool to rank genes complying with a given set of constraints.

## Results

In this section, the Population Proportion Ranking Method (PPRM) is described, followed by a discussion on the assumptions used in PPRM and results of testing of this method on simulated and experimental data.

### Population Proportion Ranking Method

Let the number of samples in class T (for 'Treated') be m_T _and the number of samples in class N (for Normal) be m_N_. T_i_, for i = 1 to m_T_, are the expression levels of a gene in the m_T _samples of class T and N_j_, for j = 1 to m_N_, are the expression levels of the gene in the m_N _samples of class N.

The inter-class variability is quantified using ratio ${\text{R}}_{\text{TN}}^{\text{i},\text{j}}$, defined below:

$${\text{R}}_{\text{TN}}^{\text{i},\text{j}}=\frac{{\text{T}}_{\text{i}}}{{\text{N}}_{\text{j}}}for\;i=1:m_{T},j=1:m_{N}$$

The intra-class variability is quantified using ratios ${\text{R}}_{\text{TT}}^{\text{i},\text{k}}$ and ${\text{R}}_{\text{NN}}^{\text{j},\text{1}}$ defined below:

$${\text{R}}_{\text{TT}}^{i,k}=\frac{{\text{T}}_{\text{i}}}{{\text{T}}_{\text{k}}}for\;i=1:m_{T},k=i+1:m_{T}$$

and

$${\text{R}}_{\text{NN}}^{\text{j},1}=\frac{{\text{N}}_{\text{i}}}{{\text{N}}_{\text{1}}}for\;j=1:m_{N},1=j+1:m_{N}$$

A ratio-cutoff is chosen based on biological knowledge of the magnitude of difference in expression level required between groups (C_TN_) and amount of variability that is acceptable within groups (C_TT _and C_NN_). For example, an inter-class ratio cutoff of 3 implies that there should be at least a 3 fold difference in expression between a sample in class T and another sample in class N for the gene to be identified as differentially expressed for that pair of samples and an intra-class ratio cutoff of 1.5 means that the maximum acceptable difference in expression between any two samples in a class is 1.5 fold. Increasing C_TN _will lead to identification of genes which have a larger magnitude of difference between the two classes, while changing intra-class ratios (C_TT _and C_NN_) allows the user to change the magnitude of variability acceptable within a given class. Naturally, since increasing C_TN _or decreasing C_TT _or C_NN _leads to a decrease in the number of genes identified as differentially expressed, these parameters can be used to identify a tractable number of differentially expressed genes of a certain nature, for further analysis.

To identify differentially expressed genes, the fraction of the inter-class ratios ${\text{R}}_{\text{TN}}^{\text{i},\text{j}}$ which are either greater than the ratio-cutoff C_TN _or smaller than 1/C_TN _is calculated as f_TN_. Similarly the fraction of intra-class ratios ${\text{R}}_{\text{TT}}^{i,k}$ and ${\text{R}}_{\text{NN}}^{\text{j},\text{1}}$ which are either greater than the ratio-cutoff C_TT _and C_NN _respectively or smaller than 1/C_TT _and 1/C_NN _respectively are calculated as f_TT _and f_NN_.

Thus,

$${\text{f}}_{\text{TN}}=\frac{\left(\begin{array}{l} {\text{Number of inter group ratios greater than C}}_{\text{TN}}\\ +\text{Number of inter group ratios smaller than }1{\text{/C}}_{\text{TN}} \end{array}\right)}{{\text{m}}_{\text{T}}*{\text{m}}_{\text{N}}}$$

$${\text{f}}_{\text{TT}}=\frac{\left(\begin{array}{l} {\text{Number of intra T group ratios greater than C}}_{\text{TT}}\\ +\text{Number of intra T group ratios smaller than }1{\text{/C}}_{\text{TT}} \end{array}\right)}{{\text{m}}_{\text{T}}*({\text{m}}_{\text{T}}-1)/2}$$

$${\text{f}}_{\text{NN}}=\frac{\left(\begin{array}{l} {\text{Number of intra N group ratios greater than C}}_{\text{NN}}\\ +\text{Number of intra N group ratios smaller than }1{\text{/C}}_{\text{NN}} \end{array}\right)}{{\text{m}}_{\text{N}}*({\text{m}}_{\text{N}}-1)/2}$$

Genes for which f_TN _is significantly greater than f_TT _and f_NN _are calculated using a standard statistical test of comparing population proportions [45]. Thus, the null hypothesis tested is f_TN _≤ f_TT _and/or f_TN _≤ f_NN_. In biological terms, this translates to a null hypothesis that the inter class variability is less than or equal to the intra class variability. The allowable inter and intra-class variability is quantified by their respective ratio cutoffs. The test statistic is calculated using the formula [45]:

$${\text{z}}_{\text{TT}}=\frac{{\text{f}}_{\text{TN}}-{\text{f}}_{\text{TT}}}{\sqrt{{\text{q}}_{\text{TT}}(1-{\text{q}}_{\text{TT}})(\frac{1}{{\text{m}}_{\text{T}}{\text{m}}_{\text{N}}}+\frac{1}{{\text{m}}_{\text{T}}({\text{m}}_{\text{T}}-1)/2})}}$$

$${\text{z}}_{\text{NN}}=\frac{{\text{f}}_{\text{TN}}-{\text{f}}_{\text{NN}}}{\sqrt{{\text{q}}_{\text{NN}}(1-{\text{q}}_{\text{NN}})(\frac{1}{{\text{m}}_{\text{T}}{\text{m}}_{\text{N}}}+\frac{1}{{\text{m}}_{\text{N}}({\text{m}}_{\text{N}}-1)/2})}}$$

where,

$${\text{q}}_{\text{TT}}=\frac{{\text{N}}_{\text{TN}}+{\text{N}}_{\text{TT}}}{{\text{m}}_{\text{T}}{\text{m}}_{\text{N}}+{\text{m}}_{\text{T}}({\text{m}}_{\text{T}}-1)/2}$$

$${\text{q}}_{\text{NN}}=\frac{{\text{N}}_{\text{TN}}+{\text{N}}_{\text{NN}}}{{\text{m}}_{\text{T}}{\text{m}}_{\text{N}}+{\text{m}}_{\text{N}}({\text{m}}_{\text{N}}-1)/2}$$

m_T _is the number of samples in class T

m_N _is the number of samples in class N

N_TN _is the number of ratios ${\text{R}}_{\text{TN}}^{\text{i},\text{j}}$ which are greater than the ratio-cutoff C_TN _or smaller than 1/C_TN_

N_TT _is the number of ratios ${\text{R}}_{\text{TT}}^{\text{i},\text{k}}$ which are greater than the ratio-cutoff C_TT _or smaller than 1/C_TT_

N_NN _is the number of ratios ${\text{R}}_{\text{NN}}^{\text{j},\text{1}}$ which are greater than the ratio-cutoff C_NN _or smaller than 1/C_NN_

The significance values p_TT _and p_NN_, corresponding to z_TT _and z_NN _are calculated. These values indicate the significance level of the difference between proportions of the inter-class ratios greater than inter-class cutoff and the respective intra-class ratios greater than intra-class cutoff. A p-value cut-off is chosen (p_cutoff_) to identify genes with significant difference between the proportion of the inter-class and intra-class ratios which are greater than the respective ratio-cutoffs chosen. Thus, genes with p_TT _< p_cutoff _and p_NN _< p_cutoff _are selected as differentially expressed. It should be noted here that the test allows the flexibility of controlling intra-class variability in only any one class or in both classes. For example, differentially expressed genes with low variability in N only can be ranked by using the condition p_NN _< p_cutoff _and a relatively stringent value of C_NN_. In summary, the three parameters which need to be chosen to rank differentially expressed genes are listed in Table 1.

**Table 1:** Parameters used in the Population Proportion Ranking Method

**Parameter**	**Description**	**Remark**
C_TN_	Ratio cutoff for inter-class ratios	Chosen based on the required magnitude of difference in expression between the two classes
C_TT_, C_NN_	Ratio cutoff for intra-class ratios	Chosen based on allowable heterogeneity in expression within a class
p_cutoff_	Significance value cutoff for significance of difference between the proportions of inter-class and intra-class populations greater than respective ratio cutoffs	Chosen based on required stringency in difference between the proportions of inter-class and intra-class populations greater than respective ratio cutoffs

#### Assumptions

The test makes an assumption of 1) Random and independent selection of inter-class and intra-class ratios and 2) Large sample size of the inter-class ratios and inter-class ratios, so the sampling distributions of differences of proportions are very closely normally distributed. Though the samples within each class are reasonably expected to be selected randomly and independently, all inter- and intra- group ratios are not independent. Specifically, there are only (m_T _+ m_N _-1) independent inter-class ratios and (m_T _-1) or (m_N _-1) independent intra-class ratios. Hence the effective sample size is smaller leading to smaller reported significance values. However, in order to capture the true variability between all samples in a group or between groups, it is essential to use all inter-class and intra-class ratios. Hence the reported significance values are not exact and should only be used to calculate the relative merit of genes, and not the actual distance between them.

### Testing

PPRM is tested on 5 sets of simulated data representing various intra and inter-class variability situations and compared to the t test, PPST, COPA, OS, ORT and MOST. PPST is implemented through the online implementation provided by Lyons-Weiler *et al*. [39] available at . COPA, OS, ORT and MOST were implemented using the R code by Lian [44] available at . PPRM is also tested on three publicly available cancer datas and used to identify predictor genes that can be used for classification. The classification accuracy using predictors identified by the PPRM is comparable to other reported classification accuracies.

### Simulated data

**Figure 2:**
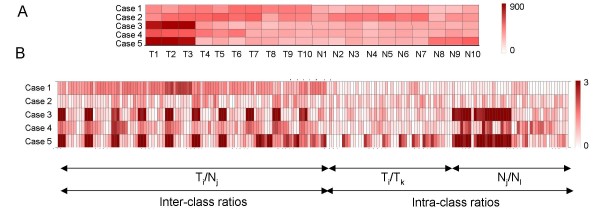
**Distribution of expression values and inter- and intra-class ratios for all 5 cases listed in Table 2 (for a representative example) (A) Heatmap of expression levels across samples in the two classes for all 5 cases, using the parameters in Table 2. **Values above 900 are indicated by the maximum intensity. (B) Heatmap of absolute values of log_2 _transformed inter-class and intra-class ratios for all 5 cases. Values above 3 are indicated by the maximum intensity.

PPRM is tested on a simulated data set of 10000 genes measured in 20 samples belonging to two classes: 10 samples in class T and 10 samples in class N. 1000 out of the 10000 genes were modeled as differentially expressed. Simulated data sets were generated using the random number generator function in Matlab (The Mathworks, Inc., Natick, MA, USA) under normal distribution. To simulate the scenario where only a subset of samples within a class are differentially expressed, in case 3, 4 and 5, it is assumed that ~30% of the samples for the 1000 genes show differential expression. Table 2 indicates the parameters for the normal distributions that were used to simulate the data (mean and standard deviation). Figure 2A shows a representative example for the distribution of expression levels across samples in the two classes for all 5 cases, using the parameters in Table 2. Figure 2B shows the distribution of inter-class and both intra-class ratios for all 5 cases. Data for non-differentially expressed genes is simulated using parameters of a mean of 100 and standard deviation of 30 (not indicated in Table 2). The inter-class ratio cutoff is chosen equal to the ratio of mean expression level in the two classes. PPST, COPA, OS, ORT, MOST and t test were also used to analyze the simulated data. For COPA, OS, ORT and MOST, p-values for each gene were calculate from the test statistics as the proportion of the 9000 genes (with an identical distribution in both classes) with absolute test statistics larger than that of this gene [43]. A significance value cutoff of 0.01 is used for all methods.

**Table 2:** Parameters used to generate simulated data for the 5 cases tested

	**Class T**			**Class N**		
	
	**Number of Samples**	**Mean**	Stdev*	**Number of Samples**	**Mean**	Stdev*
Case 1	10	250	50	10	100	30
Case 2	10	250	100	10	200	100
Case 3	3	900	100	10	100	30
	7	100	50			
Case 4	3	400	50	5	100	30
	3	300	50	5	130	30
	4	100	30			
Case 5	3	900	100	7	100	30
	7	100	50	3	400	100

* Standard deviation

Parameters for the normal distributions used are indicated only for the 1000 differentially expressed genes. Data for the 9000 non differentially expressed genes is simulated using a mean of 100 and standard deviation of 30. Expression levels of samples for each case (for a representative example) are indicated in Figure 2A in the form of a heatmap

For all the methods, the following metrics were used to evaluate the performance of the method.

$$\text{Recall}=\frac{\text{True positives}}{\text{True positives}+\text{False negatives}}\times 100$$

$$\text{FPR}=\frac{\text{False positives}}{\text{False positives}+\text{True negatives}}\times 100$$

where:

True positives = Number of truly differentially expressed genes identified

False positives = Number of genes identified which are not differentially expressed

False negatives = Number of truly differentially expressed genes not identified

True negatives = Number of genes which are not differentially expressed, which are correctly not identified

FPR = False positive rate

**Figure 3:**
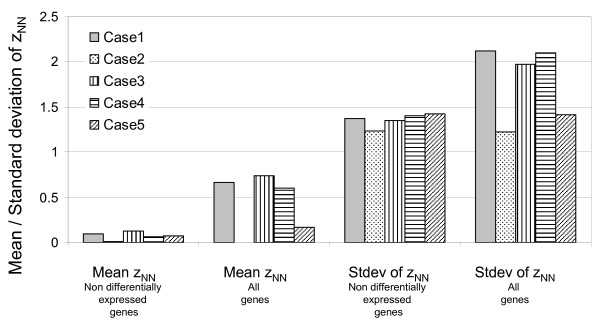
**Mean and standard distribution of z_NN _for all 5 cases of simulated data indicated in Table 2.** The mean and standard deviation of only the 9000 non-differentially expressed genes is indicated separately from the mean and standard deviation of all 10000 genes. Case 1: Solid bars, Case 2: Dotted fill, Case 3: Vertical lines, Case 4: Horizontal lines, Case 5: Diagonal lines.

In order to assess the effect of violation of the assumption of independence, the distributions of z_TT _and z_NN _were analyzed for the simulated data. The mean and standard distribution of z_NN _for all 5 cases analyzed is shown in Figure 3. The average of the means for the 9000 non- differentially expressed genes across all 5 cases is 0.07, and of the standard deviation is 1.3, while the values of the same statistics for all 10000 genes across all 5 cases are 0.4 and 1.8 respectively. The reader is reminded that due to lack of independence of all the inter- and intra- group ratios, the p-values calculated are not exact and are to be used only for the purpose of prioritizing and ranking genes. In all the following discussion, the p-value cutoff is used for selecting a subset of the top ranked differentially expressed genes. An alternate approach would be to select a fixed number of top ranking genes. However, in cases where more than one gene has the same significance value, selecting a fixed number of top ranking genes involves randomly disregarding some genes. Hence to avoid this, the p-value cutoff approach is used.

**Figure 4:**
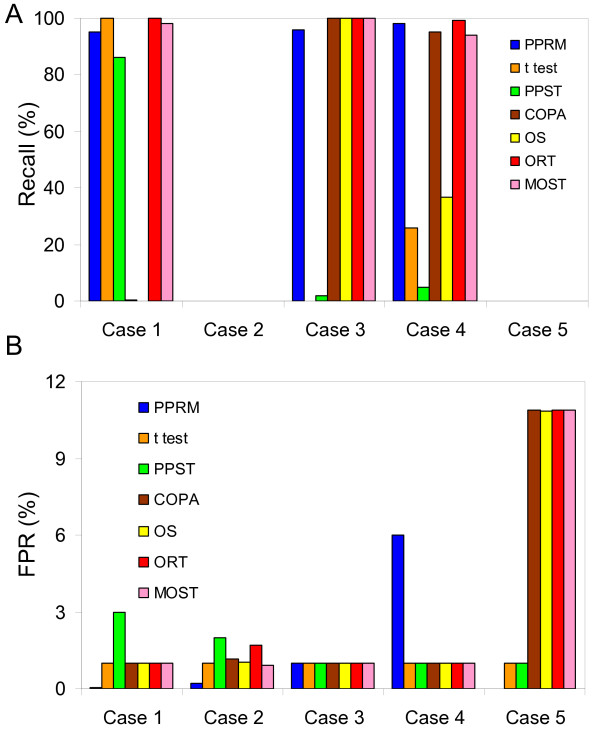
**Results on the analysis of simulated data using PPRM, t test, PPST, COPA, OS, ORT and MOST.** (A) Percentage Recall for all 5 cases listed in Table 2 (B) Percentage FPR for all 5 cases listed in Table 2. PPRM: Blue, t test: Orange, PPST: Green, COPA: Brown, OS: Yellow, ORT: Red, MOST: Pink.

An ideal method will have a 100% Recall and 0% False Positive Rate (FPR). Figure 4 summarizes the Recall and FPR for all methods for the 5 cases described in Table 2. The inter-class ratio cutoff (C_TN_) used is chosen based on the known ratio of the means of all samples in the two classes. The intra-class ratio cutoffs (C_TT _and C_NN_) are chosen to be equal to the inter-class cutoff in all cases, with exceptions as described below. The C_TN_, C_TT _and C_NN _values used for PPRM in for all 5 cases are listed in Table 3. A significance value cutoff of 0.01 is used for all methods.

**Table 3:** Inter-class and intra-class ratio cutoffs used in the analysis of simulated data using PPRM

	**C_TN_**	**C_TT_**	**C_NN_**
Case 1	3	3	3
Case 2	1	1	1
Case 3	3	-	3
Case 4	2	-	2
Case 5	2	2	2

Case 1 is an example of a case of differential expression, with low variability within samples. As seen in Figure 2B, all intra-class ratios have small values while the inter-class ratios are higher. PPRM, t test, ORT and MOST identify most differentially expressed genes, with PPRM having the lowest FPR.

Case 2 is an example of genes which do not have a significant difference in expression level in the two classes and have larger variability as compared to case 1. Here again, PPRM has the lowest FPR among all methods tested.

Case 3 is an example of genes which have a low variability in one class, but very high variability in the other due to a subset of samples. Here, the intra-class ratios for class T are small, while those for class N are high (Figure 2B). In this case, COPA, OS, ORT and MOST have a 100% Recall. PPRM does not identify any differentially expressed gene when heterogeneity in both classes is controlled (i.e. both conditions p_TT _< p_cutoff _and p_NN _< p_cutoff _used; data not shown). However, if heterogeneity in class T is allowed by only using the condition p_NN _< p_cutoff_, PPRM has a 96% Recall and 1% FPR, which is similar to the other methods. This is an example of the application of PPRM allowing the control of heterogeneity in any one class only.

Case 4 is an example of genes which have moderate variability in one class and high variability in the other. This is different from case 3 in having the magnitude of expression level between the two classes lower (average 2-fold) than that in case 3 (average 3-fold). Again, the t test, PPST and OS have a poor Recall. ORT and MOST have a Recall of 99% and 94% with a FPR of 1%. PPRM does not identify any differentially expressed gene when heterogeneity in both classes is controlled (data not shown), but when heterogeneity in class T is allowed (p_NN _< p_cutoff _is the only condition used), a 98% Recall is obtained, but at the cost of 6% FPR. There is thus a trade-off between identifying all truly differentially expressed genes and obtaining false positives. Increasing the stringency of the parameters (e.g. increase in C_TN_, decrease in p_cutoff_) can reduce FPR at the expense of Recall (data not shown).

Case 5 is an example of a gene with high variability in both classes, which should ideally not be identified as differentially expressed. Here, there does not appear to be a significant difference in the distribution of inter-class and intra-class ratios, as seen in Figure 2B. PPRM has FPR of 0.02% which is the lowest, followed by the t test and PPST at 1%. COPA, OS, ORT and MOST have a FPR of 11%. (Note: Not accounting for variability in class N by PPRM has a FPR of 7%. This FPR decreases as the values of C_TN _and C_TT _are increased)

In summary, in cases where the heterogeneity in the sample population is low as exemplified by Case 1, all tests except COPA and OS perform reasonably well in identifying true positives. The t test, PPST, COPA and OS fail to identify differentially expressed genes in most cases, whereas PPRM, ORT and MOST can identify most differentially expressed genes in all cases. However, though ORT and MOST give lower FPR for case 4, they give higher FPRs than PPRM in Case 2 and 5 representing non differentially expressed genes.

In the case of simulated data, the inter-class and intra-class ratios were chosen based on knowledge of expression levels of truly differentially expressed genes, which will clearly not be the case in real world data. However, for real-world data, these parameters will be chosen based on the requirement of specific types of genes. More than one set of parameters can be used for an analysis to obtain different groups of differentially expressed genes. For example, using low intra-class cutoffs allows the identification of differentially expressed genes with low intra-class variability whereas using a higher value of one intra-class cutoff (C_TT _or C_NN_) also identifies genes with higher heterogeneity in that group (T or N, respectively).

### Experimental Data

Variability in simulated data cannot mimic the heterogeneity in real biological data, and hence PPRM is also tested on the following three publicly available experimental data sets. Since there is no gold standard of a list of differentially expressed genes in real world data, simply identifying differentially expressed genes in a data set is not adequate to test the method. Though the distinguishing feature of PPRM lies in its ability to identify differentially expressed genes with greater variability between samples in a class, the method is also able to identify differentially expressed with low variability within groups based on the choice of parameters used for the test. Hence, in analyzing real biological data, an approach of identifying a relatively small number of 'predictor' genes is adopted and their accuracy in being able to predict the class of an unknown sample is tested. This approach of validation of new methods of identification of differentially expressed genes has also been used by other researchers [38]. The classification accuracy is expected to be similar to other reported values, but not necessarily better since the primary goal of this report is not to identify genes for classification.

In order to identify biomarkers, stringent conditions are used (i.e. higher values of inter-class ratio cut-off, lower value of intra-class ratio cutoff and lower values of cutoff of the p-value) to select a small number of genes with low heterogeneity in expression within a class. For the biological data sets used below, misclassification rates reported using some other methods are included for the sake of general comparison. For the leukemia data set, the independent data set available is used to test the prediction power of selected genes. For all other data sets, a LOOCV technique is used. To avoid bias in gene selection from the sample which is left out, the list of differentially expressed genes is calculated separately every time with the same parameters, and this list is used to predict the class of the sample that is left out. Classification is performed using Discriminant Analysis in Matlab (The Mathworks, Inc., Natick, MA, USA).

#### Leukemia data

Gene expression profiles of two types of leukemia samples were derived from 47 patients with acute lymphoblastic leukemia (ALL) and 25 patients with acute myeloblastic leukemia by Golub *et al *[18]. Data is obtained from the Broad Institute website at .

The training data consists of gene expression data from 27 patients with acute lymphoblastic leukemia (ALL) and 11 patients with acute myeloblastic leukemia (AML) while the independent data set consisted of 20 ALL samples and 14 AML samples. Genes for which less than 5 samples had a "Present" call were not used in the analysis. The values of the three parameters for PPRM are listed in Table 4. In the original publication by Golub *et al *[18], the authors identified 50 genes as biomarkers based on their method of neighborhood analysis, and tested the use of these genes to predict the class of samples in the independent data set. They correctly classified all samples on which a prediction is made, 29 out of 34, declining to predict the other five. Using a support vector machine method, Furey *et al *[49] could correctly classify 30 to 32 out of the 34 samples. Using the parameters listed in Table 4, six differentially expressed genes were identified using PPRM. These genes were used as biomarkers to test the accuracy of class prediction for samples in the independent data set. Out of the 34 samples, 33 were accurately classified using the 6 genes identified by PPRM.

**Table 4:** Parameters used for the analysis of the three cancer data sets

**Parameter**	**Leukemia**	**Prostate Cancer**	**Colon cancer**
C_TN_	2	3.5	3
C_TT_	1.5	2	3
C_NN_	1.5	2	-
p_cutoff_	0.0001	0.001	1e-10

#### Prostate cancer data

The prostate cancer data set generated by Singh *et al *[48] consists of 92 samples, 45 of which were non-tumor prostate samples and 47 of which were prostate tumor. The data set is publicly available and is obtained from the Broad Institute website . Genes for which less than 20 samples had a "Present" call were not used in the analysis. A LOOCV technique is used for this data set. In the original paper, a 10% error rate in sample classification using LOOCV is obtained, while Dettling *et al *[50] reported misclassification rates between 5%–14% using supervised clustering. In this study, using the parameters listed in Table 4, an 8% error rate in sample classification using LOOCV is obtained. The number of biomarker genes identified in all LOOCV runs is between 9 and 18.

#### Colon cancer data

The colon cancer data set generated by Alon *et al*. [47]consists of 62 samples, 40 tumor samples and 22 normal controls. The gene expression data were downloaded from . LOOCV is also used for this data set. Other researchers have obtained misclassification rates (including unclassified samples) between 8% to 34% [50-54] using various methods like nearest neighbor classifiers, SVM, boosting, 'Minimum Redundancy- Maximum Relevancy', Bayes error filter for gene selection and supervised clustering.

In this study, using the parameters listed in Table 4, a 16% error rate in sample classification using the LOOCV is obtained. The number of biomarker genes identified in all LOOCV validation runs is between 7 and 13, with one exception where 23 genes were identified.

## Discussion

DNA microarray analysis is being increasingly used to identify differences between two or more classes like diseased and healthy tissue. Most methods used for the identification of differentially expressed genes between two classes identify genes where the variability between samples in a class is low. However there can be significant variability among samples in a class due to differences between individual subjects and their environment [36]. PPRM uses inter-sample ratios to quantify variability in expression. This method allows for the identification of genes where the user can define the allowable heterogeneity within one or both classes and required difference in expression between samples in the two classes. Since all inter-class and intra-class ratios used in this method are not independent, the significance values calculated by PPRM are not exact and should be used only for ranking and prioritizing genes. The mean and standard deviation of the test statistic are reported for the simulated data sets to facilitate the assessment of the impact of violation of the assumptions for the sample size of 10 samples in each class (i.e. 100 inter-class ratios and 45 intra-class ratios for each class, out of which 19 and 9 respectively are independent).

PPRM works as well or better than all other methods tested in data sets where the heterogeneity in samples is low. In simulated cases tested where variability is high, ORT, MOST and PPRM successfully identify most differentially expressed genes. In addition to a high Recall, it is necessary for any method to minimize the number of false positives identified. Genes with high variability in expression levels among samples in both classes should not be identified as differentially expressed simply because the expression level in some samples in one class is different than the expression level of some samples in the other class. This is tested in case 2 and 5 in the simulated data, where reassuringly a very low FPR of 0.2 and 0.02% is obtained using PPRM. However, for these cases ORT and MOST consistently resulted in higher values of the test statistic for the 1000 non differentially expressed genes resulting in high FPRs. This is likely due to the lack of an additional constraint of relative difference in these methods as available in PPRM.

PPRM is also able to identify differentially expressed with low variability within groups, based on the choice of parameters used for the test. Hence, it is possible to test it on publicly available cancer data sets by assessing the success of the genes identified in correctly classifying samples in the two groups. The classification accuracies obtained for the three publicly available cancer data sets used for testing are similar to those reported using other methods.

## Conclusion

The Population Proportion Ranking Method (PPRM) presented here quantifies variability in terms of inter-sample ratios and allows for the identification of genes where the user can define the allowable heterogeneity within one or both classes and required difference in expression between samples in the two classes for ranking differentially expressed genes.
